# Immunomodulatory Polysaccharide from *Chlorophytum borivilianum* Roots

**DOI:** 10.1093/ecam/neq012

**Published:** 2011-01-09

**Authors:** Mayank Thakur, Paul Connellan, Myrna A. Deseo, Carol Morris, Vinod K. Dixit

**Affiliations:** ^1^Centre for Phytochemistry and Pharmacology, Southern Cross University, Lismore, P.O. Box 157, Lismore, NSW 2480, Australia; ^2^Department of Pharmaceutical Sciences, Dr H. S. Gour University, Sagar, India

## Abstract

*Chlorophytum borivilianum* Santapau & Fernandes (Liliaceae) is an ayurvedic *Rasayana* herb with immunostimulating properties. The polysaccharide fraction (CBP) derived from hot water extraction of *C. borivilianum* (CB), comprising of *∼*31% inulin-type fructans and *∼*25% acetylated mannans (of hot water-soluble extract), was evaluated for its effect on natural killer (NK) cell activity (*in vitro*). Human peripheral blood mononuclear cells (PBMCs), isolated from whole blood on a Ficoll-Hypaque density gradient, were tested in the presence or absence of varying concentrations of each *C. borivilianum* fraction for modulation of NK cell cytotoxic activity toward K562 cells. Preliminary cytotoxicity evaluation against P388 cells was performed to establish non-cytotoxic concentrations of the different fractions. Testing showed the observed significant stimulation of NK cell activity to be due to the CBP of *C. borivilianum*. Furthermore, *in vivo* evaluation carried out on Wistar strain albino rats for humoral response to sheep red blood cells (SRBCs) and immunoglobulin-level determination using enzyme-linked immunosorbent assay (ELISA), exhibited an effectiveness of *C. borivilianum* aqueous extract in improving immune function. Present results provide useful information for understanding the role of CBP in modulating immune function.

## 1. Introduction

*Chlorophytum borivilianum* constitutes an important class of ayurvedic herbs which are known as *Rasayana*, that is, herbs with immunostimulatory and adaptogenic properties [1]. “*Rasayan*a" therapy is a specialized measure in Ayurveda that deals with the proper modulation of the neuro-endocrino-immunological system [2]. *Rasayan* therapy aims to boost and nourish the body to balance the bodily functions and counteract the aging process by bringing homeostasis, that is, a balance between the various bodily humors (*viz. the Vata, the Pitta and the Kapha*) [3–5]. Evidence collected on various *Rasayan*a herbs signifies their utility as immunomodulators with low toxicity [6, 7].

*Chlorophytum borivilianum* Sant F. (Fam. Liliaceae) is widely cultivated throughout India, especially in the states of Madhya Pradesh, Andhra Pradesh and Maharashtra, and exported globally for its medicinal usage [8]. Major phytochemical components reported from the roots of *C. borivilianum* include steroidal saponins, fructans and fructoligosaccharides (FOS), acetylated mannans, phenolic compounds and proteins [9–12]. The plant is acclaimed for various health benefits, which include its ability to ameliorate hyperlipidemia, diabetes-induced sexual dysfunction and prevent heat-induced testicular damage. The plant is also considered to be useful as an antioxidant [13–16].

In our previous study, we reported the improvement of *in vivo* immunomodulation by the ethanol extract of *C. borivilianum* [17]. Extensive phytochemical evaluation of the roots of *C. borivilianum* led to the quantification of nearly 60% w/w of polysaccharides in the hot water-soluble material, which were found to be mainly fructans and acetylated mannans [16]. Since these polysaccharides have been cited for their immunomodulatory potential, various fractions of *C. borivilianum* aqueous extract were evaluated for their effect on the modulation or enhancement of human natural killer (NK) cell activity, hemagglutination (HA) titer values against sheep red blood cells (SRBCs) and immunoglobulin G (IgG) levels in rats.

## 2. Methods

### 2.1. Plant Material

**Figure 1:**
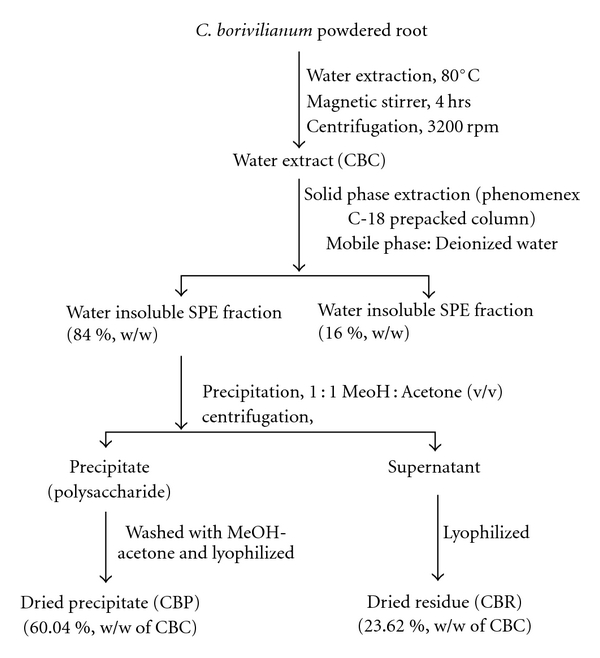
Fractionation of crude aqueous extract and various fractions evaluated for biological activity.

Dried roots of *C. borivilianum* were procured from Nandan Agro Farms, Hyderabad, India, and identified at the Department of Pharmaceutical Sciences, Dr. H.S. Gour Vishwavidyalaya, Sagar (India). A voucher specimen of the same has been deposited in the departmental herbarium (MTCB-1608). The powdered roots were subjected to hot water extraction using the methodology reported previously [15] and the procedure on the isolation and fractionation of the aqueous extract of *C. borivilianum* has been detailed in Figure 1. Cytotoxicity of the fractions was evaluated on the P388 cell line. The aqueous extract of the root powder (CBC), the polysaccharide (CBP) and non-polysaccharide (CBR) fractions were used for evaluating the effect on modulation of NK cell activity *in vitro*, and HA titer assay and IgG level *in vivo.*

### 2.2. Animal Group

A total of 72 Wistar strain albino rats (either sex) in the weight range of 160–180 g were fed a standard pellet diet and water *ad libitum*. The animals were housed at room temperature (24 ± 2°C) on a normal day-night cycle (6.00 a.m. to 6.00 p.m.). The guidelines given by the Committee for the Purpose of Supervision and Control of Experiments on Animals, India, were strictly followed. SRBCs were procured from the Haffkine Biopharmaceutical, Mumbai, India. All the other chemicals and reagents used were of analytical grade.

### 2.3. Animal Group for HA Titer Value

Fourteen days prior to experimentation, two sets of six groups, comprising of six animals in each group, received experimental drug administration as per the following schedule.

- Group I: Served as control and was administered vehicle only.
- Group II: 100 mg of *C. borivilianum* aqueous extract per kilogram of body weight (CBC).
- Group III: 50 mg of *C. borivilianum* polysaccharide fraction per kilogram of body weight (CBP50).
- Group IV: 100 mg of *C. borivilianum* polysaccharide fraction per kilogram of body weight (CBP100).
- Group V: 50 mg of *C. borivilianum* non-polysaccharide fraction per kilogram of body weight (CBR 50).
- Group VI: 100 mg of *C. borivilianum* non-polysaccharide fraction per kilogram of body weight (CBR 100).

### 2.4. HA Titer Determination

On day 0, paralleling the above mentioned treatment, animals were immunized by injecting 0.5 mL of 5.0 × 10^9^ SRBC/mL via the intraperitoneal route (i.p.). On day 7 the animals were challenged by injecting the same volume of SRBCs. Blood samples were collected by retro-orbital puncture on day 14 for antibody titer. Hemagglutination antibody titer was determined by using the micro-titration technique described by Damre et al. [18]. For experimentation, 40 *μ*L of 0.1% (w/v) bovine serum albumin (BSA) solution in normal saline was pipetted into the wells of micro-titration plates. To this solution, 40 *μ*L of serum of either the treated or control animal was added, which was later serially diluted 2-fold. Further 20 *μ*L of a 0.1% suspension of SRBCs in BSA-saline was added to each well; the plate was initially incubated at 37°C for 60 min followed by 60 min at 4°C. The value of the highest serum dilution causing visible hemagglutination was considered to be the antibody titer.

### 2.5. IgG-Level Determination

For the determination of IgG levels (on day 0) animals were immunized by injecting 0.2 mL of 1% w/v BSA in phosphate-buffered saline (PBS) solution. After immunization, each group of animals was subjected to a drug-treatment schedule of 14 days as described above. On day 7, blood samples were collected and the IgG level of immunized animals was measured using a simple indirect enzyme-linked immunosorbent assay (ELISA) and recorded as primary antibody levels. On day 14, animals were further challenged with 0.2 mL of 1% w/v BSA. On day 21, blood samples were collected by retro-orbital puncture, subjected to an ELISA determination of IgG levels and recorded as secondary antibody levels [19]. To perform the ELISA, wells of the ELISA plates were coated with 100 *μ*L of 1% w/v BSA in PBS and incubated at 37°C for 1 h. In order to ensure the removal of unbound BSA, three washings were done using PBS-0.05% Tween (PBS-T) solution. Serum samples were diluted 1000-fold in PBS and 25 *μ*L of the diluted serum samples were added in corresponding wells, followed by 1 h incubation. The unbound antibodies were removed by washing thrice with PBS-T solution. A solution of 50 *μ*L of rabbit anti-rat IgG-Horseradish peroxidase (HRP) was added to all the wells and incubated for 60 min. All wells were washed 3 times to remove the unbound materials and 50 *μ*L of substrate tetramethylbenzidine-hydrogen peroxide (TMB-H_2_O_2_) was added and incubated for 5 min. The enzyme-substrate reaction was terminated by the addition of 50 *μ*L of 5N sulfuric acid (H_2_SO_4_). The absorbance was measured at 450 nm and the results calculated were recorded and expressed as log_10_ values.

### 2.6. Cellular Cytotoxicity Evaluation

Mouse leukemic P388 cells were routinely cultured in a humidified 5% CO_2_ incubator (Sanyo, Japan) in Dulbecco's Modified Eagle's Medium (DMEM) + 10% horse serum (HS), to which penicillin G (500 U/mL), streptomycin (5000 *μ*g/mL) and 3.5 mg/mL of D-glucose at 37°C were added. Cell concentrations were measured using an AcT Diff analyzer (Beckman, Australia). To maintain exponential growth, cells were seeded at 1 × 10^5^ cells/mL and passaged every 4-5 days. Chlorambucil and curcumin were used as positive controls for the assay. The commercially available ATPLite kit (Perkin Elmer, USA) was used to assess cell viability. A final cell concentration of 4000 cells/well was maintained using appropriate dilutions in DMEM + 10% HS media.

Two-fold serial dilutions of chlorambucil and curcumin were made starting at concentrations of 600 and 100 *μ*g/mL, respectively. Solvent and media controls were also included in the assay. The samples were assayed at various concentrations of CBC, CBP and CBR starting at 10 mg/mL. Testing was performed in duplicate. For this, 50 *μ*L of sample was added to each well along with 50 *μ*L of P388 cell suspension. The cells and samples were incubated at 37°C for 24 h in a humidified 5% CO_2_ incubator (Sanyo, Japan). Measurement of cell proliferation (ATP) was performed as per ATPLite kit protocol (Perkin Elmer, Netherlands) using the Micro Beta 1450 plate reader (Perkin Elmer, USA).

### 2.7. NK Cell Activity

Samples (CBC, CBP and CBR) were prepared by dissolving the material at an initial concentration of 10 mg/mL in de-ionized Milli Q water (Millipore, UK) and appropriately diluted in water to obtain the final concentrations as required. The samples were vortexed and sonicated for 10 min to ensure complete solubility.

*In vitro* NK cell activity was assayed by flow cytometry using a Becton Dickinson FACS Calibur instrument. The methodology reported by Standen et al. [20] was used with little modification. In brief, peripheral blood mononuclear cells (PBMCs) were prepared from fresh, whole, lithium-heparinized blood using Ficoll-Hypaque (Amersham Biosciences). Human PBMCs were suspended in advanced Roswell Park Memorial Institute (RPMI)-1640 medium (2% fetal BS; 1% l-glutamine; 2% penicillin/streptomycin) and aliquots of cells were pre-incubated with each extract dilution for 2 h at 37°C in 5% CO_2_. The PBMCs (NK cells/effectors) were then incubated for 2 h (37°C, 5% CO2) with target cells (K562; ATCC) pre-labeled with Vybrant DiO cell labeling solution (Molecular Probes, Invitrogen, V-22886), a green fluorescent dye that allows differentiation of target from effector cells. The effector : target cell ratio used was 30:1. Propidium iodide (Molecular Probes, P-3566), a red fluorescent DNA dye, was added following incubation to label target cells, which were rendered permeable by NK cell activity. A target cell control (no effectors) was run for each sample to monitor spontaneous target cell death. Protein-bound polysaccharide (PSK), which has been validated for its selective activation of NK cells in a mouse experimental tumor model, was used as a positive control [21]. A solvent control (water) was also run and each sample was tested in duplicate. The percentage of dead target cells was determined by flow cytometry using CellQuest Pro software. The percentage of specific cytotoxicity was determined by subtracting the percentage of dead cells in the target control tube from the percentage of dead target cells in each test sample.

### 2.8. Statistics

Statistical analysis and sample size was determined using Instat v 2.01. Confidence was set at 95%. All the groups were compared to control using one-way Analysis of Variance (ANOVA) followed by Dunnet's test. Significance was set at *P* < .05.

## 3. Results

Effectiveness of *C. borivilianum* on various aspects of the immune system was clearly demonstrated in the present study. CBC, CBP and CBR were evaluated for their effect on NK cell activity, humoral and cellular immune response.

### 3.1. Cytotoxicity Evaluation

**Figure 2:**
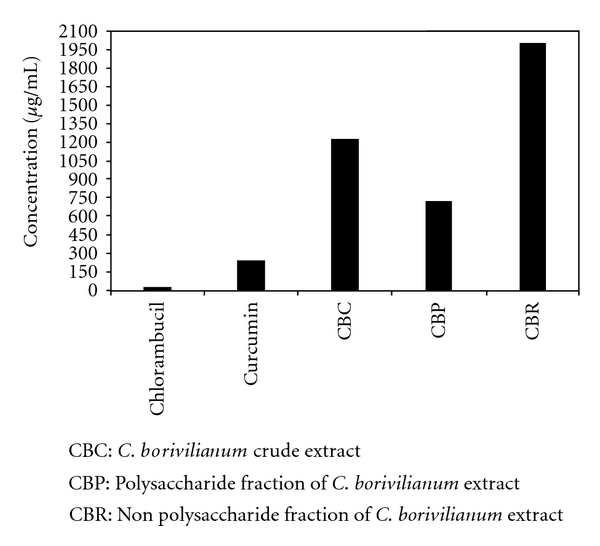
IC_50_ values of *C. borivilianum* aqueous extract and fractions against P388 cells.

As previously reported, CBC was found to be non-toxic up to a dose of 2 g per kilogram of body weight [16]. In the present study, an evaluation of the cytotoxicity and determination of IC_50_ was carried out on the sensitive mouse leukemic P388 cell line. Curcumin and chlorambucil were used as positive controls for the determination of cytotoxicity. The results confirmed the non-toxic nature of the *C. borivilianum* extract under investigation. CBC and CBP exhibited IC_50_ values of 1219.67 and 722.31 *μ*g/mL, respectively (Figure 2).

### 3.2. HA Titer Value

**Figure 3:**
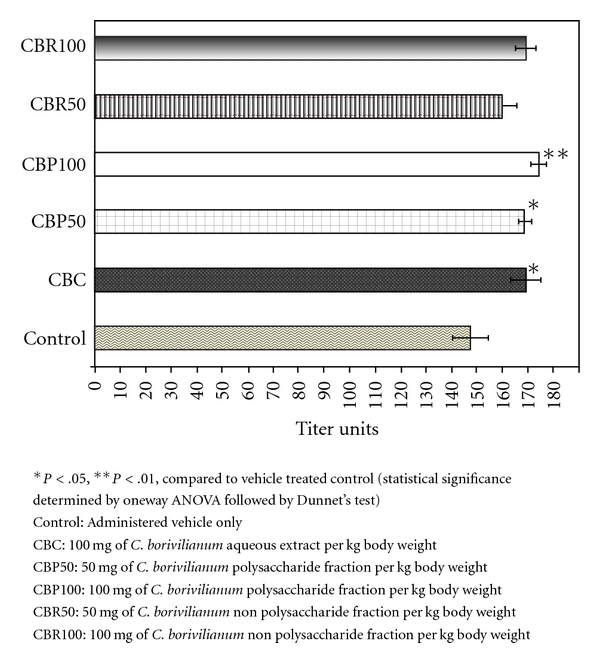
Effect of *C. borivilianum* aqueous extract and fractions on HA titer values in Wistar rats.

The values for HA titer in the case of the vehicle-treated control group animals were established at 147.6 ± 11.9. Significantly higher HA titer values of 169.2 ± 6.1, 174.3 ± 3.1, and 168.6 ± 2.6 were observed for CBC and CBP at 100 *μ*g/mL and CBP at 50 *μ*g/mL, respectively. However, CBR only showed mild increases in HA titer of *∼*8.6% and 8.1% at 100 and 50 *μ*g/mL, respectively. The results for HA titer values observed are detailed in Figure 3.

### 3.3. IgG Level

**Figure 4:**
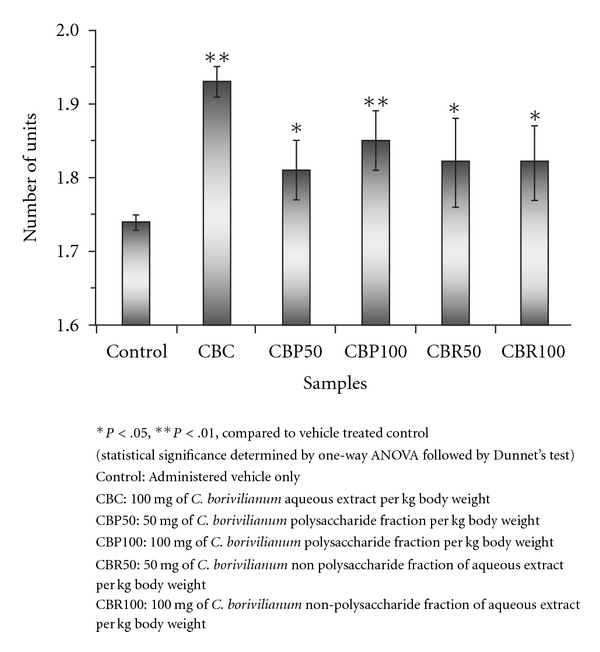
Effect of treatment with *C. borivilianum* aqueous extract and fractions on Immunoglobulin (IgG) levels in Wistar rats after 28 days.

ELISA-based measurements were used for the secondary antibody responses in immunized animals. Administration of CBC resulted in a significant increase (*P* < .01) in IgG level (1.93 ± 0.02), followed by CBP100 (1.85 ± 0.01) and CBP50 (1.81 ± 0.04). CBR50 and CBR100 gave similar IgG levels of 1.82 ± 0.12 and 1.82 ± 0.11 units, respectively. In the case of control animals (vehicle only), the value was found to be 1.74 ± 0.013 units (Figure 4).

### 3.4. NK Cell Activity

**Figure 5:**
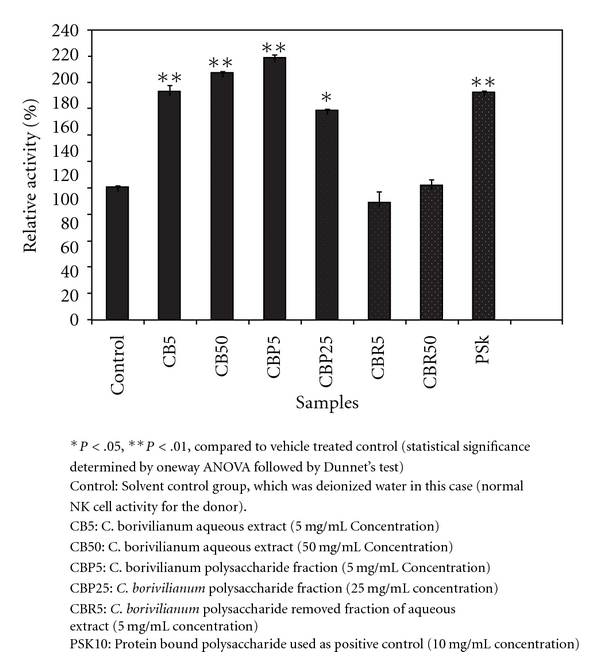
Effect of *C. borivilianum* aqueous extract and fractions on *in vitro* NK cell activity against K562 cells.

In comparison to the control, a significantly higher effect of CBP (*P* < .01) in augmenting the NK cell activity was observed. Although CBC stimulated NK cells significantly (*P* < .05), most of the effect appeared to be contributed by CBP alone. Therefore, polysaccharides of *C. borivilianum* appeared to be solely responsible for NK cell augmentation. In the case of CBR, no effect was observable, and the result obtained was equivalent to that of the solvent control group. At a final concentration of 5 *μ*g/mL the CBP fraction resulted in an *∼*2-fold increase in NK cell activity (98 ± 2.5%), whereas at 25 *μ*g/mL, the % increment was 58.4 ± 0.3%. This indicates that the isolated polysaccharide fraction is more effective at the lower concentration tested. In the case of CBC, some semblance of a dose-dependent effect was observed, wherein a 72.9 ± 4.4% and 86.6 ± 0.7% increase in NK cell activity was observed with CBC at 5 and 50 *μ*g/mL, respectively. There was no enhancement in relative NK cell activity for CBR at 5 and 25 *μ*g/mL. The differences in relative activity compared to control groups have been shown in Figure 5.

## 4. Discussion

Ayurvedic *Rasayan* herbs have been acclaimed for their ability to enhance the functionality of the immune system. Appropriate modulation of biological homeostasis in order to boost the ability to fight infection, counteract diseases, prevent cancer, and so forth, is the primary focus of *Rasayan* therapy [22]. *C. borivilianum* is renowned for its ability to boost immune function [17]. Interactions among immune cells and those of immune cells with the other tissues in the body are highly diversified and mostly unexplored. The ability to counteract infections or to fight against cancer mainly involves a three-tiered functionality: humoral immunity, cellular immunity and the regulators of immune system, such as cytokines [23]. Previously we had reported the effectiveness of ethanol fraction of the *C. borivilianum* extract on improving non-specific immunity [17]; in the present study we have been able to determine the possible role of CBP on immune modulation.

A burgeoning area of research is the development or discovery of immunomodulatory agents that are free from toxic side effects and can be used for a long duration, thus resulting in continuous immuno-activation [24]. Previous research carried out on *Rasayan* herbs has validated that they activate immune functionality without causing an imbalance in overall physiology; this aspect has been excellently reviewed by Vayalil et al. [6]. Similar to many immunomodulatory substances, *Rasayan*as are not directly cytotoxic to tumor cells; rather they produce significant inhibitory effect on ascites tumor development and solid tumor growth in mice during the treatment period [25]. *Rasayan*as have been previously considered to act via *in vivo* augmentation of NK cell activity as well as antibody-dependent cellular toxicity. In the present study, an enhanced NK cell activity of CBP as well as CBC against K562 myeloid leukemic cells clearly validates the immunopotentiating ability of fructans and mannans isolated from *C. borivilianum* [26].

Augmentation of NK cell activity is considered an important parameter for improved immunological function. NK cells are a subset of lymphocytes that are important in the body's defense against viral infections and malignancy, participating in innate immunity and early defenses [26, 27]. They are defined functionally by their ability to mediate spontaneous cytotoxicity, lysing a broad range of target cells without prior sensitization and without restriction by major histocompatibility complex (MHC) antigens [28, 29]. Impaired NK cell activity is associated with increased sensitivity to infection [30]. NK cells are a major force in counteracting, and fighting, against cancer.

As previously mentioned, *Rasayan* herbs are generally regarded as non-toxic even at high doses. A toxicity assay using the ATPLite kit clearly suggested that the extracts and fractions from *C. borivilianum* were non-cytotoxic against P388 cells, a sensitive mouse cell line. In our previous studies on the plant, *in vivo* toxicity was evaluated and the extracts were found to be non-toxic even at a dose of 2 g per kilogram of body weight. Lack of cytotoxicity is an important attribute of *Rasayan* drugs and this was validated in the case of *C. borivilianum* as well.

**Figure 6:**
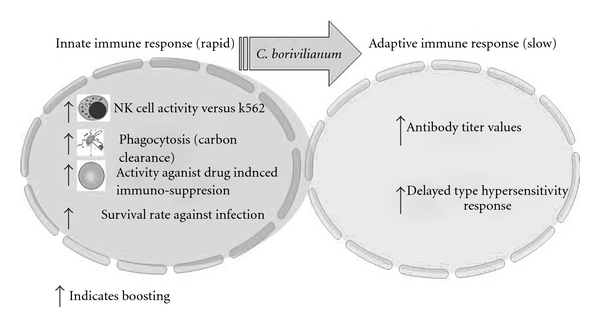
A diagram summarizing the overall effects of *C. borivilianum* extract on innate and adaptive immune responses.

The crude *C. borivilianum* extract as well as the polysaccharide fraction were able to enhance the antibody titer, while the non-polysaccharide fraction was effective in enhancing a cell-mediated response. Augmentation of the humoral response was evidenced by increased antibody production in response to SRBC challenge in the post-immunization drug treatment. The enhanced responsiveness is indicative of up-regulation of macrophages, dendritic cells and B-lymphocyte subsets involved in antibody synthesis. T-lymphocyte activation is also considered an important attribute of polysaccharides, which is directly correlated with immuno-stimulation. This cascade of functionalities provides evidence for an enhancement of humoral as well cellular immune responsiveness [31]. A diagrammatic representation of the overall assessment of *C. borivilianum* is shown in Figure 6.

*Rasayan*a herbs or their extracts have been found to be effective in enhancing the production of cytokines such as interleukin (IL)-2, interferon (IFN)-*γ* and granulocyte macrophage colony-stimulating factor (GM-CSF) in albino rats [32]. Further studies in this direction are warranted on the extracts/fractions of *C. borivilianum*.

In recent years, there have been reports on the presence of specific polysaccharides, such as fructans, acetylated mannans, xylans and glucans, in *Rasayan* herbs. These polysaccharides from medicinal plants exhibit biological activities of importance for improving human health. Perhaps the most important activity would be on the immune system, which may lead to the production of nutritional supplements in cancer treatment [33, 34]. As validated in the case of *C. borivilianum*, the use of these polysaccharides will stimulate the immune system and may also contribute to lowering the dose of existing immune therapeutics. These polysaccharides can be used in relatively large doses with no side effects; accompanying these facets are the juxtaposed benevolent attributes such as their effects on different viral infections, diabetes and aging-related problems [35, 36].

The present study provides further insight into the potential immunomodulator herb *C. borivilianum* and provides some *in vitro* evidence. It can be further inferred from this study that the polysaccharide fraction is the most effective in augmenting the NK cell activity as well as humoral immunity. The remaining fraction of extract, which is rich in phenolics and saponins [13], was useful in improving the HA titer, but had no effect on NK cell activity. Thus, it can be concluded that the *C. borivilianum* extract acts via a cascade of mechanisms, modulating the immune system to improve and restore a healthy state. The polysaccharides from *C. borivilianum* could be considered for cancer treatment via post-chemotherapy revival of the immune system. This study further validates and provides molecular evidence for the potential benefits of *Rasayan* herbs of the ayurvedic system of medicine.

## Funding

MT would like to thank the Department of Education, Employment and Workplace Relations (DEEWR) for the funding in the form of Endeavour Postdoctoral Research Fellowship.
